# Lactoferrin: A glycoprotein that plays an active role in human health

**DOI:** 10.3389/fnut.2022.1018336

**Published:** 2023-01-05

**Authors:** Xiang Cao, Yang Ren, Qinyue Lu, Kun Wang, Yanni Wu, YuHao Wang, Yihui Zhang, Xiang-shun Cui, Zhangping Yang, Zhi Chen

**Affiliations:** ^1^College of Animal Science and Technology, Yangzhou University, Yangzhou, China; ^2^Department of Animal Science, Laboratory of Animal Developmental Biology, Chungbuk National University, Cheongju, Republic of Korea; ^3^Joint International Research Laboratory of Agriculture and Agri-Product Safety, Ministry of Education, Yangzhou University, Yangzhou, China; ^4^International Joint Research Laboratory in Universities of Jiangsu Province of China for Domestic Animal Germplasm Resources and Genetic Improvement, Yangzhou, China

**Keywords:** lactoferrin, antimicrobial, anticancer, immunoregulation, expression regulation

## Abstract

Lactoferrin (Lf), existing widely in human and mammalian milk, is a multifunctional glycoprotein with many functions, such as immune regulation, anti-inflammation, antibacterial, antiviral, and antioxidant. These extensive functions largely attribute to its ability to chelate iron and interfere with the cellular receptors of pathogenic microorganisms and their hosts. Moreover, it is non-toxic and has good compatibility with other supplements. Thus, Lf has been widely used in food nutrition, drug carriers, biotechnology, and feed development. Although Lf has been continuously explored and studied, a more comprehensive and systematic compendium is still required. This review presents the recent advances in the structure and physicochemical properties of Lf as well as clinical studies on human diseases, with the aim of providing a reference for further research of Lf and the development of its related functional products.

## 1. Introduction

Human beings use lactoferrin (Lf) for more time than we can imagine. We often see the word lactoferrin on the packaging of dairy products now, but do you know? As early as the war in Europe, knights used whey from colostrum to clean wounds caused by bows, arrows, and swords to avoid worsening infection. Lactoferrin was first discovered in breast milk by scientist Johanson Bengt in 1960 (1). Then scientists discovered the protein in the body fluids and various cells of some organisms and began a lot of research. In 1971, Rümke et al first found that the content of lactoferrin in serum protein of cancer patients was higher (2). This phenomenon was later found in many types of cancer, and gradually lactoferrin became a sign of cancer. In 1994, Mitra first reported the expression of human lactoferrin in plant cells, and subsequently recombinant human lactoferrin was successfully expressed in a variety of plants including tobacco, potato, rice, tomato, and carrot (3). In 2002, Van Berkel et al. pioneered the construction of transgenic cows expressing high levels of recombinant human lactoferrin, which yielded 0.4–3.0 g/L. The recombinant human lactoferrin was found to have similar biological activity to the natural protein (4). Through the large-scale production of recombinant human lactoferrin, human lactoferrin will have important implications in functional food and healthcare.

Lactoferrin, a kind of iron-bound multifunctional cationic glycoproteins (5), belongs to the transferrin (TF) family together with serum transferrin, ovotransferrin, melanin ferritin, and carbonic anhydrase inhibitor (6). Mainly existing in mammalian milk, it is also distributed in tears, nasal and bronchial secretions, saliva, bile, and other body fluids (7). In addition, Lf is also produced by hematopoietic tissue of bone marrow and is present in neutrophil granules. The composition of milk varies considerably between species, because physiologically and structurally, lactation is designed to meet the nutritional requirements of newborns of a particular species. The content of lactoferrin in breast milk and bovine milk also varies significantly. In donkey milk, lactoferrin accounts for 4.48% of the total whey protein, and its molecular weight is about 75ku. Its content in donkey milk is about 0.4 mg/mL, which is between cow milk and human milk. Although bovine lactoferrin (bLf) shares more than 70% homology with human lactoferrin (hLf), the concentration of hLf colostrum is higher, at about 7 g/L. Currently, the versatility of Lf in food and other industries has led to a surge in its demand and a promising application in the market. It has been added to many commercial products, such as nutritional supplements, infant formula, cosmetics, and toothpaste (8). However, the research on the structure, physical and chemical properties of Lf still lacks systematic introduction and prospect (9). This is what this review will especially explore.

**FIGURE 1:**
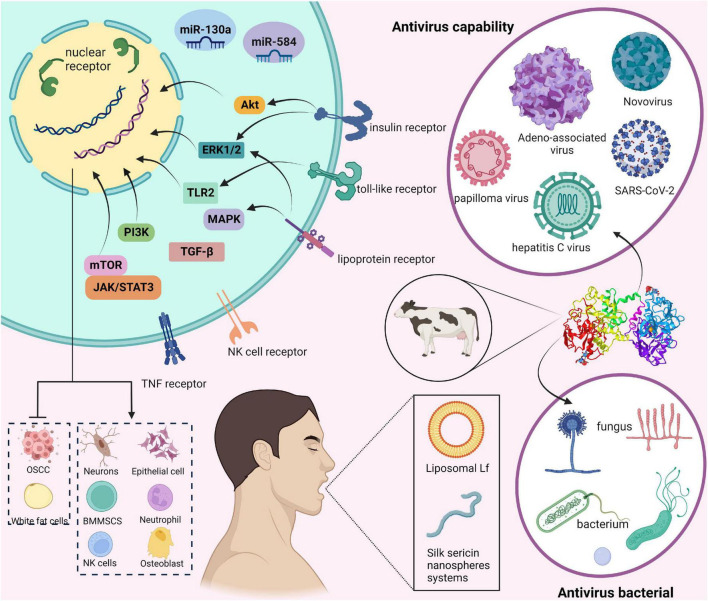
Overview of this review of lactoferrin (Lf).

In humans and animals, Lf is mainly found in the products of exocrine glands located at the gateways of the digestive, respiratory, and reproductive systems (10). This suggests Lf plays a role in non-specific defense against invading pathogens and is the first line of defense against microbial and viral infections (11). In addition, in plasma, Lf is derived from neutrophils which degranulate and synthesize Lf during the inflammatory process. In general, the expression of the Lf gene is influenced by a variety of factors and has multiple expression specificities (12). Over the past 60 years, Lf has been extensively studied, and its role in numerous biological functions has been accepted by the scientific community. These days Lf is used as a biomarker for COVID-19 (13), inflammatory bowel disease (IBD) (14), aging (15), and neurological disorders (16). Furthermore, Lf has been shown to be involved in a variety of physiological and protective effects. Some of the most studied to date include antimicrobial activity, antiviral, cell proliferation and differentiation, cytokine regulation, inflammatory response, and immunomodulatory activity (17–19). However, the cellular and molecular mechanisms by which Lf regulates inflammatory, antiviral, and immune responses have not been fully elucidated. Hence, this paper reviews the main characteristics and major biological functions of Lf, and particularly clarify the mechanism of action of using exogenous Lf as a therapeutic agent for human diseases to identify new research prospects. We summarize the layout of this paper in Figure 1.

## 2. Structure of lactoferrin

**FIGURE 2:**
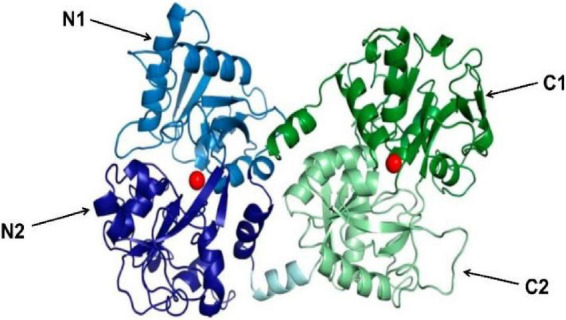
3D diagram of bovine lactoferrin [adapted from Fabiana Superti et al. (103)]. N-lobe is blue (N1 is light blue, N2 is dark blue), C-lobe is green (C1 is dark green, C2 is light green). The peptide bonds linking N-lobe and C-lobe are expressed in light cyan. The Iron ion is a red sphere.

Lactoferrin, a cationic iron-binding glycoprotein, consists of a single polypeptide chain (approximately 700 amino acid residues) with a relative molecular mass of around 80 ku (20). In humans, mice, and bovine, Lf homology is high. Among them, the homology reaches 70% between humans and mice, 69% between humans and bovine, and 63% between mice and bovine (21). The secondary structure of Lf is mainly composed of alternating arrangements of α-helix and β-fold. On the basis of it, there is a tertiary structure composed of a polypeptide chain according to the three-dimensional structures of hLf solutions and crystals measured by X-rays. This structure folds into two symmetrical spherical lobes with similar structures, namely the N-lobe and the C-lobe (Figure 2). The N-lobe corresponds to the amino acid residues 1-333 in the peptide chain, while the C-lobe corresponds to the amino acid residues 345-692. These two lobes can be further divided into two regions, respectively, N-1 (amino acids 1-90, 251-333) and N-2 (amino acids 91-250), C-1 (amino acids 345-431, 593-689) and C-2 (amino acids 432-592). In addition, the N-lobe and C-lobe are connected by a small peptide bond (amino acids 334-344), forming the overall structure as “two Ginkgo biloba type,” with each lobe having an iron-binding site in the cleft between the N1 and N2, C1 and C2 sub-structural domains. What’s more, iron ions are covalently linked to four amino acid residues (two tyrosine, one aspartate and one histidine) of Lf (22). In the presence of carbonate, each lobe is able to reversibly bind Fe^3+^ with a maximum binding amount of 1.4 mg Fe/g protein. In addition to Fe^3+^, some other metal ions such as Zn^2+^, Cu^2+^, and Mn^3+^ can also be bound with Lf.

According to its iron saturation, Lf can be classified into iron-deficient lactoferrin (Apo-Lf) (iron saturation is below 5%) and iron-saturated lactoferrin (Holo-Lf) (iron saturation is above 85%). The denaturation temperatures of these two Lfs are 60–66 and 88–92°C, respectively. In addition, they have different tertiary structures: Apo-LF is characterized by an “open” N-lobe and a “closed” C-lobe, whereas both lobes of Holo-LF are “closed,” thus having a more compact structure than iron-free Lf and iron-binding Lf. The iron saturation of Lf is closely associated with its physicochemical properties. For example, iron-free Lf has stronger antibacterial and antioxidant activities than iron-saturated lactoferrin. Furthermore, the osteogenic activity of Lf decreases with the increase of iron saturation *in vitro* and *in vivo* (23). Moreover, the isoelectric point of Lf, a basic protein, fluctuates depending on the degree of iron binding and glycosylation.

The isoelectric point of bLf is about 8.0 and that of hLf is about 6.0. As an important regulator of free iron levels in mammalian body fluids, Lf can bind iron with high affinity and maintain it at a low pH value, thus providing proteins with antibacterial and antioxidant properties (24). The retention rate of iron in bLf is as high as 85% when the environmental pH of Lf is 6.5, but only 10% when the environmental pH is 2.0 (25). The solubility, foaminess, and emulsification properties of bLf are improved after high pressure processing. Bellamy et al. first isolated a segment of bLf from the N-terminal polypeptide called bovine lactoferrin active polypeptide (LfcinB), which was hydrolyzed by pepsin under acidic conditions. LfcinB, derived from amino acids 17-41 of bLf, is an antibacterial peptide containing 25 amino acid residues, including 5 Trp, 3 Lys, and several aromatic amino acid residues. Being strongly basic (pH > 8.15), it can be released in the digestive tract under normal physiological conditions. In addition, LfcinB has a hydrophilic lipid structure with two reverse β-folded structures in aqueous solution, which is heat-resistant and not easily degraded in the digestive tract. More importantly, it exhibits higher antibacterial and anticancer activities than natural Lf.

## 3. Physicochemical properties and applications of lactoferrin

As mentioned above, the broad functions of Lf can be attributed to its ability to chelate iron and interfere with the cellular receptors of pathogenic microorganisms and their hosts. Moreover, the function of Lf is inextricably linked to its own structure and the surrounding environment. Recently, many studies have indicated Lf has potential emerging applications in the clinical treatment of human diseases, agricultural production, and daily use industries. This section summarizes and sorts out the physicochemical properties and applications of Lf.

### 3.1. Physicochemical properties of lactoferrin: Antibacterial, antiviral, and anti-inflammatory

Lactoferrin has a high affinity for ferric ions, which is 250–300 times higher than that of TF. Acting as an iron-redox homeostasis (Fe-R-H) regulator, Lf, chelating iron and neutralizing iron-mediated free radicals, reduces oxidative stress and improves host defense by optimizing iron metabolism (26). Lf mainly transports iron ions in the intestine, so it plays a crucial role in the absorption of iron ions in the intestine of mammals. Cellular uptake of iron has a negative feedback regulation. When intracellular iron is deficient, the LfRs on the cell surface subsequently increase and so does the cellular uptake of Lf.

**FIGURE 3:**
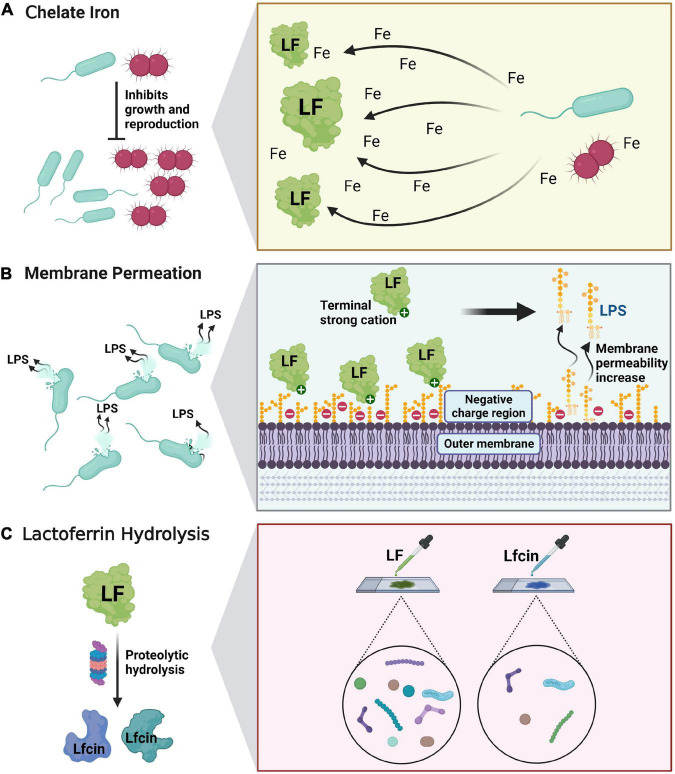
Antibacterial and bactericidal mechanism of lactoferrin. **(A)** Lactoferrin acts as an antibacterial agent by chelating free iron-mediated activity. **(B)** Lactoferrin increases the permeability of bacterial cell membranes through the strong cation-binding region at the amino terminus, producing a direct bactericidal effect. **(C)** Lf hydrolyzes to obtain antimicrobial peptide, which exerts stronger antimicrobial effect.

Lactoferrin exerts antibacterial effects against a wide range of microorganisms including bacteria, fungi, viruses and parasites through three different mechanisms: (a) Bacteriostatic activity mediated by chelating free iron, which is essential for bacterial growth and proliferation, i.e., “taking away” bacterial nutrients (27). (b) Lf increases the permeability of bacterial cell membrane through the strong cation-binding region at the amino terminus, causing the exudation of bacterial lipopolysaccharide and other cell contents from the outer membrane, thus producing a direct bactericidal effect. (c) Lf hydrolyzes to obtain antimicrobial peptide, which exerts stronger antimicrobial effect (Figure 3) (28). In 1972, Bullen et al. first suggested that Lf has an antibacterial effect which is dependent on iron concentration (29). Using immunofluorescence, Arnold et al. found that apo-Lf could bind to the surface of the bacterium and detach external nutrients, thus preventing them from entering the bacterium and causing their death (30). A prospective randomized trial showed that in 60 women with bacterial vaginitis (BV), the incidence of BV decreased after vaginal administration of 100 mg and 200 mg of Lf for 10 days. Furthermore, the vaginal microbiota of patients with BV altered and proportion of Lactobacillus increased accordingly (31).

**FIGURE 4:**
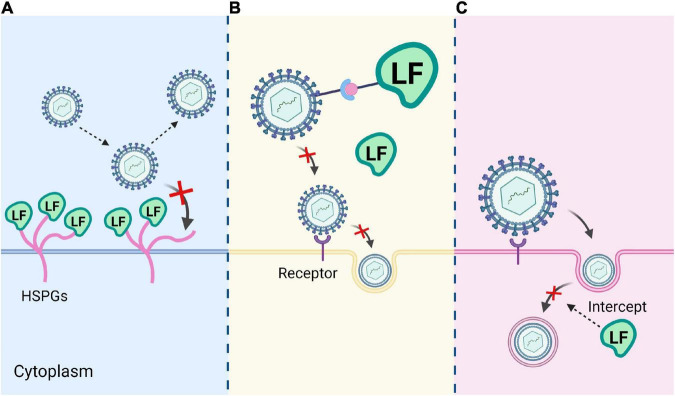
Three mechanisms of Lf antivirus. **(A)** Lf binds to heparan sulfate proteoglycans (HSPGs) on the host cell surface. **(B)** Lf binds directly to viral proteins and inhibits viral adsorption to target cells. **(C)** Lf interferes with intracellular transport of the virus and blocks the transmission of the viral genome to the cytoplasm.

Lactoferrin also has great potential in the antiviral aspect. So far, studies have shown that Lf is effective against human papilloma virus (HPV), herpes simplex virus 1 (HSV-1), herpes simplex virus 2 (HSV-2), cytomegalovirus (CMV), Human Immunodeficiency Virus (HIV), hepatitis B virus (HBV), hepatitis C virus (HCV), respiratory syncytial virus (RSV), hantaan virus (HV), rotavirus (RV), poliovirus (PV), adenovirus (AdV), and the currently spreading SARS-CoV-2 in *in vitro* and *in vivo* experiments (32). In several cell lines, Lf is shown to block the attachment of SARS-CoV-2 virus to cytosolic heparan sulfate and enhance the interferon response (33). *In vitro* experiments, Campione et al. demonstrated that Lf could exert its antiviral activity against SARS-CoV-2 by directly attaching to SARS-CoV-2 and the surface components of recipient cells (34). SARS-CoV-2 infects ACE2-expressing cells and enters the cells through direct plasma membrane fusion or endocytosis. The antiviral mechanisms of Lf include (a) binding to heparan sulfate proteoglycans (HSPGs) on the surface of host cell, thereby reducing viral attachment and subsequent viral entry; (b) binding directly to viral proteins to inhibit viral adsorption to target cells; and (c) interfering with intracellular transport of virus and intercepting the delivery of viral genomes to the cytoplasm (Figure 4). Lf from different sources may show different antiviral and antibacterial activities. Studies have shown that camel lactoferrin (cLf) inhibits hepatitis C virus (HCV) more than human lactoferrin (hLf), bovine lactoferrin (bLf) and sheep lactoferrin (sLf), respectively (35). This may be related to the iron saturation of Lf, as apo-Lf has a higher antiviral potency than holo-Lf.

Lactoferrin is a modulator of mammalian innate and acquired immunity, which relies on its ability to bind to conserved structures of pathogens. Lf can be converted into an anti-inflammatory molecule capable of protecting the host from harmful immune responses. Specifically, Lf is immunomodulated by interacting with specific receptors on immune cells such as lymphocytes, monocytes, dendritic cells, macrophages, natural killer cells (NK), etc. Lf interacts with specific receptors on these immune cells. By inhibiting activation of P38 MAPK derived from TLR4 in microglia, bovine lactoferrin (bLf) restrains IL-18 production, thus ameliorating neuropathic pain in mice (36). Oral administration of liposomal bovine lactoferrin (LbLf) effectively prevents the progression of rheumatoid arthritis (RA) in mice by inhibiting tumor necrosis factor (TNF-α) production in the pancreas (37). In addition, Fan et al. found that apo-LF, compared to holo-LF, could significantly attenuate LPS-induced inflammatory bowel injury by modulating the PPAR-γ/PFKFB3/NF-κB inflammatory pathway (38).

### 3.2. Application in the treatment of human diseases

Lactoferrin has been used in clinical treatment for decades. Since 2020, with the spread of COVID-19, it has been reported that Lf, as a non-toxic, broadly functional “wonder protein,” has great potential in preventing SARS-COV-2 infection and improving patients’ immunity (39). Increased free iron and hyperferritinemia in patients with COVID-19 due to iron dysregulation and overload may exacerbate the inflammatory process through oxidative damage to cellular biomolecules induced by reactive oxygen species (ROS) (13). Facing with a large amount of “cellular debris,” Lf can bind to free iron ions to achieve antioxidant effects, and the antioxidant activity is negatively correlated with iron saturation. Meanwhile, bLf is effective against different variant strains of SARS-COV-2, including South African B.1.351, British B.1.1.7, Brazilian P.1, and Indian Delta variants (40).

#### 3.2.1. Lf and chronic diseases

Chronic diseases, especially cardiovascular disease (CD), diabetes and osteoporosis, have become the focal point of global public health. Khan and co-workers found that the antidiabetic properties of camel milk may be due to the interaction between bLf and the insulin receptor (IR), thereby inducing IR phosphorylation and activating AKT and ERK1/2 signaling pathways (41). Blood pressure is the most important factor in cardiovascular health, and Lf is a good source of orally active antihypertensive peptides (42). The main target of anti-hypertensive food-derived peptides is angiotensin-I-converting enzyme (ACE). After a series of experiments, Ruiz and co-workers found that LfcinB can inhibit ACE activity in mice and reduce systolic blood pressure in spontaneously hypertensive rats (43). In addition, studies have shown that Lfcin significantly prolongs prothrombin kinase and prothrombin time, which is an important entry point for the treatment and prevention of thrombosis (44).

Meanwhile, Lf, a novel bone growth factor, plays a physiological role in bone growth and healing. To be specific, Lf induces the proliferation and differentiation of osteoblasts and inhibits osteoclast formation in bone anabolic activity (45). Guo et al. demonstrated the osteogenic properties of Lf *in vivo* for the first time and found that Lf has a therapeutic effect on postmenopausal osteoporosis in rats (46). A human whole-transcriptome microarray study, which differentially elucidates the coding and non-coding transcript expression profiles and functional network analysis of human bone marrow mesenchymal stem cells (BMSCs) during osteogenic differentiation, revealed that signaling pathways such as MAPK signaling pathway, TGF-β signaling pathway and Toll-like receptor are associated with osteogenic differentiation of BMSCs (47). Besides, Lf binds to low-density lipoprotein receptor-related protein 1 (LRP1) on the surface of osteoblasts and regulates the ERK1/2 pathway, thus promoting the proliferation of osteoblasts. Lf also induces the activation of p42/44 MAPK signaling in primary osteoblasts. It is reported that LfcinB enables the eradication of *Bacillus purpureus* and the reduction of *Staphylococcus aureus* in diabetic mouse wounds. It can also promote migration of keratinocyte and angiogenesis in diabetic mice, being conductive to diabetic wound healing.

#### 3.2.2. Lf and neurodegenerative diseases

Iron imbalance and oxidative stress (OS) are common in patients with neurodegenerative diseases, such as Parkinson’s disease (PD), Alzheimer’s disease (AD), dementia, depression, and multiple sclerosis (MS) (48). Due to the blood-brain barrier (BBB), most central nervous system drugs cannot enter the brain. However, Lf can rapidly cross the blood-brain barrier *via* receptor-mediated transcytosis and accumulate in brain capillary endothelial cells. Lactoferrin receptors (LfRs) are present on glioma cells, brain microvessels, and neurons. By binding to specific lipoprotein receptors, Lf regulates neuronal differentiation to stimulate the intracellular phosphatidylinositol kinase (PI3K) and extracellular signal-regulated kinase (ERK) transduction pathways, thus upregulating neuron-specific enolase (NSE) expression and promoting neuronal cell differentiation (49). Sokolov et al. found that salivary and tear lactoferrin levels are significantly higher in PD patients than in controls (non-PD patients), and the plasma Lf levels are inversely correlated with the severity of PD (50). Nevertheless, it is noteworthy that although Alzheimer’s patients have the same neurodegenerative disease, their salivary Lf levels are significantly lower compared to controls (non-AD patients). Researchers have explored receptor-mediated approaches to target drug and non-drug molecules to the brain, thereby achieving exogenous gene expression across a wide range of brain regions. Gothwal et al. coupled Lf to polyamidoamine generation 3.0 (PAMAM G3.0) dendrimers to deliver rivastigmine (RIV) to the mouse brain, thus significantly enhancing acetylcholine levels, and improving overall memory in mice drastically (51).

#### 3.2.3. Lf and gastrointestinal health

While inhibiting the growth of harmful intestinal bacteria, Lf promotes the growth of beneficial bacteria such as Lactobacillus acidophilus and *Bifidobacterium bifidum*, thereby maintaining intestinal health by regulating the balance of intestinal flora. A study of 48 term and preterm infants found that the number of fecal bifidobacteria and lactobacilli correlates significantly with fecal Lf concentrations 3 days after delivery, suggesting that Lf may promote the growth of beneficial intestinal microbiota (52). Although Lf inhibits the growth of harmful bacteria, some researchers have found large amounts of Lf in the resected stomachs of patients with gastritis, speculating that *H. pylori* takes up iron through specific Lf receptors, and more importantly, Lf may play a major role in the virulent effects of *H. pylori* infection (53).

On the other hand, Lf is used as a biomarker for inflammatory bowel disease along with fecal calprotectin and serum C-reactive protein in clinical care (54). Compared to patients with inactive inflammation and irritable bowel syndrome (IBS), patients with ulcerative colitis or Crohn’s disease have higher levels of Lf in stool and serum. A double-blind randomized clinical trial in Japanese patients with colorectal polyps showed that bLf suppresses colorectal polyps by enhancing immune responsiveness. To be specific, the polyp growth is significantly inhibited in patients with colorectal polyps who consumed 3.0 g bLf per day 1 year later. Meanwhile, serum hLf levels also increase (enhanced neutrophil activity), along with enhanced systemic NK cell activity and reinforced polyps with CD4^+^ and CD161^+^ cells (55).

Additionally, Lf also plays a role in establishing the gut microbiota in newborns. A series of randomized clinical trials were conducted to test the supplementation of enteral bLf, results of which demonstrated a significant reduction in late-onset sepsis (LOS) in preterm or low birth weight infants (56, 57). In another large randomized controlled trial, 2,203 preterm infants (less than 32 weeks) with gestation from 37 hospitals in the United Kingdom were randomly assigned to receive either bLf or an equivalent dose of sucrose placebo until 34 weeks of gestation. Surprisingly, the results showed that enteral supplementation with bLf does not reduce the risk of LOS infection in very preterm infants (58), which is also supported by subsequent studies (59, 60). Studies also indicate that LfcinB is conductive in maintaining and repairing intestinal mucosal tissues and preventing intestinal infection caused by rotavirus (61). For example, in a clinical trial in Japan, researchers found that intake of LfcinB could inhibite human norovirus infection (62).

#### 3.2.4. Lf and cancer cells

A variety of cancer cells are significantly damaged by Lf action, and it has been shown that Lf leads to cell cycle arrest, cytoskeleton damage and apoptosis induction, as well as cell migration reduction through regulation of cell cycle-associated proteins (63). Chea and co-workers reported that bLf selectively inhibits proliferation and induces apoptosis in oral squamous cell carcinoma (OSCC) cells *via* the mTOR/S6K and JAK/STAT3 pathways (64). In the assay, bLf inhibited phosphorylation of the growth and survival-associated kinases p65 and Akt in OSCC cells, which in turn inhibited activation of the STAT3 signaling pathway and induced G1/S cell cycle arrest, ultimately contributing to OSCC apoptosis. The combination of Lf and linolenic acid, on the one hand, activates the AMPK/JNK-related apoptotic pathway to inhibit growth of colorectal tumor. On the other hand, inhibition of JAK2/STAT3-related pathway suppressed the growth of xenograft esophageal tumors in mice (65).

#### 3.2.5. Absorption and utilization of Lf

The most common Lf on the market is bovine lactoferrin (BLf), which, as a bioactive and nutritional health protein, is non-toxic to animals. Currently, its global production is about 280 tons, of which Lf is ≥95% pure. Another common product of Lf is dairy-derived Lf, which is obtained by freeze-drying and spray-drying after being extracted from skim milk or whey cheese by ion exchange and ultrafiltration separation. The realization of Lf function is highly dependent on its structural integrity. To a large extent, oral Lf is digested in the stomach by gastric juices with only 1% utilization rate, and only trace amounts of intact Lf reach the small intestine (66). Researchers have also developed liposomal bovine lactoferrin (LbLf), a multilayer phospholipid vesicle containing a large number of Lf molecules, which can ensure effective Lf activity during transport. Ishikado and co-workers found that liposomal lactoferrin (LbLf) could improve the resistance of bLf to artificial gastric juice digestion (67). Lf is also incorporated into hyaluronic acid-coated liposomes and then homogenized under high pressure. *In vitro* and *in vivo* experiments found that it could reverse the symptoms of dry eye and anti-inflammatory properties without causing eye irritation (68). Xu and coworkers developed a recombinant human lactoferrin (rhLf) transgenic silk for the fabrication of silk sericin nanospheres systems (SS-NS-rhLf). When ambient pH ≥ 5.5, the negatively charged silk gliadin achieves specific targeting of SS-NS-rhLf to the positively charged colonic sites (14).

### 3.3. Application of lactoferrin in other industries

In agricultural production, the abuse of antibiotics leads to the development of drug-resistant pathogens and drug residues in the body, posing a great threat to human health and public health. BLf can be used as a new animal-derived antimicrobial agent with superior properties such as low residue, non-toxicity and non-resistance, and can be used as a dietary supplement for livestock, poultry and fish. Hu et al. found that early Lf intervention could modulate the composition of the colonic microbiota and improve the intestinal function of suckling pigs (69). Shao et al. reported that supplementation of 100 mg/kg/day of LfcinB in the diet of lactating dairy goats could reduce oxidative stress and inhibit mammary gland inflammation (70). Hashem et al. found that Lf could improve the immune characteristics and antioxidant status of *Oreochromis niloticus* when added to the diet, showing a better improvement than hyoscine (71). On the whole, it can be seen that Lf is a dietary supplement with excellent properties, playing positive effects on immune regulation, disease resistance and antioxidant in animals.

Lactoferrin is generally recognized as safe (GRAS) by the United States Food and Drug Administration (FDA). Approved by the U.S. Department of State in 2002, activated lactoferrin (aLf) can be sprayed on carcasses to prevent bacterial contamination during processing, or applied to the base or finished beef surface prior to final packaging to inhibit bacterial growth and extend shelf life (72). Using 0.5% Lf solution as a disinfectant has 90–100% killing effect on tobacco mosaic virus after spraying (73). Kobayashi et al. reported that the addition of Lf can effectively improve sperm activity and prolong the survival time of frozen-thawed semen after thawing, because Lf maintains the integrity of sperm plasma membrane and reduces the level of reactive oxygen species (ROS) (74). This has also been reported in horses (75), goats (76), and porcine (77).

**FIGURE 5:**
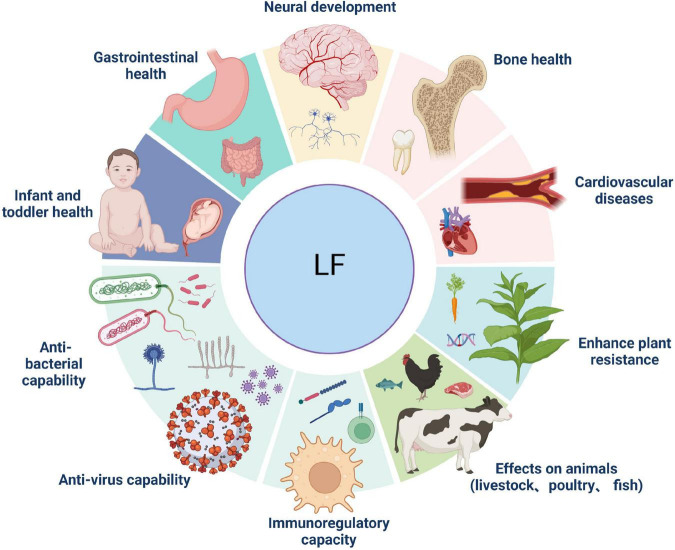
Applications of lactoferrin in the treatment of human diseases and other industries.

Above discusses the role of Lf as an exogenous substance in dietary additives, food processing, and sperm quality maintenance. What is the result of inserting Lf genes into plants and animals using transgenic technology? In 1994, Mitra et al. first reported the introduction of hLf gene into tobacco and other plants to obtain transgenic plants by Agrobacterium tumefaciens-mediated method (3). These transgenic plants showed superior antibacterial and disease resistance as well as iron content. Li et al. first created hLf transgenic dairy cattle by microinjecting 150 kb bacterial artificial chromosome (BAC) containing the entire hLf gene into bovine fetal fibroblasts *via* plasmid encoding marker gene (78). Afterward, hLf transgenic dairy goats were born on demand. It has been shown that neo-lactoferrin, carrying the full-length hLf gene, is a natural combination of recombinant hLf (90%) and goat lactoferrin (10%) isolated from the milk of transgenic goats, which can enhance IL-1β production. Moreover, Neo-lactoferrin saturated with iron ions increases the synthesis of the pro-inflammatory cytokine TNF-α (Figure 5).

## 4. What factors influence the regulation of lactoferrin gene expression?

The expression of Lf gene is influenced by many factors, and Lf was first identified as an estrogen-sensitive protein (79). To date, researchers have conducted a series of studies on the mechanisms of lactoferrin gene expression regulation in terms of its structure, nuclear receptors, transcription factors, and external factors such as nutrition.

The Lf gene is species-specific, and at least 60 Lf gene sequences have been identified in 11 mammalian species, of which hLf and bLf share 67% primary structure homology. The hLf gene is located on human chromosome 3p21.3 (80), the bLf gene is localized on chromosome 22 and U12 (81), and the rat Lf gene is localized on chromosome 9 (82) (Table 1). Researchers have cloned lactoferrin gene from a number of mammals, including humans, murine, bovine and porcine. It is found that all these genes are highly conserved and consist of 17 exons with cDNA lengths ranging from 23 to 35 kb. Studies of the promoter region of the lactoferrin gene reveal that the basic promoter of the mouse Lf gene contains a non-canonical TATA box, GATAAA and AP1/CEBP element. Near the promoter are GC-rich sequences that can bind to Sp1, Sp3, and IKLF. Bioinformatics analysis showed that the transcription factor binding sites in −2000 bp promoter region of bLf are concentrated in the −1300 to −1800 and −200 to −600 regions. Interestingly, there are substantial differences in DNA sequences among different individuals in the human population (83). Teng et al. investigated restriction endonuclease polymorphisms of the hLf gene and established common SNPs of the lactoferrin gene in 91 healthy blood donors from different races (including European, American, African, and Asian). As a result, 60 SNPs are detected in the Lf gene (promoter, exons, and introns). In addition, Asians and Africans have a higher proportion of pure-hybrid variants in amino acids 11, 382 and 523 (84).

**TABLE 1:** Composition of lactoferrin genes in human, bovine, murine, and porcine.

Lf gene	Human	Gorilla	Bovine	Murine	Porcine	Goat
Location	3q21-q23	3q21-q23	22q, U12	9q	2q12	Related locus in the bovine U12 haplogroup
Length of CDS	2136bp	2136bp	2351bp	3054bp	2578bp	2411bp
Bonding components	Sp1, TATA-box, ERE, ERRE	TATA-box, ERE, ERRE	Sp1, TATA-box	Sp1, TATA-box, GA-taaA, AP1/CEBP	Sp1, TATA-box	Sp1, TATA-box, GA-taaA, AP1/CEBP
Transcription factors	Sp1, Ets, PU.1, C/EBP(a and ε), CDP/Cut, KLF5	Sp1, Ets, PU.1, /EBP(a and ε), CDP/Cut, KLF5	Sp1, C/EBP(a and ε), CDP/Cut	Sp1, C/EBP(a and ε), CDP/Cut	C/EBP(a and ε), CDP/Cut	Sp1, C/EBP(a and ε), CDP/Cut
References	(80, 92, 104)	(80, 92, 105)	(81, 106)	(12, 82, 107)	(108, 109)	(110, 111)

Lactoferrin is present in a variety of tissues and is specifically recognized by the cellular components in tissues. Overall, lactoferrin genes are complexly regulated in different tissues in multiple ways to achieve different biological activities. This is largely dependent on lactoferrin receptors (LfRs) on the surface of specific target cells, and LfRs attach to the cell membrane surface relying on glycophosphatidylinositol and bind to different target cells to exert their respective effects (85). In 1979, Cox and coworkers first identified the presence of Lf receptors (LfRs) in the human small intestine (86), and in addition, receptors at the mRNA level for transporter Lf were also expressed in brain, heart, and liver tissues (Table 2). The concentrations of Lf in the fallopian tubes, uterus, vagina, vas deferens or epididymis are much higher than that of serum, while the Lf concentrations in the ovaries, testes, seminal vesicles and prostate are comparable to that of serum (87). The Lf gene is classical estrogen target gene, and the regulation of its expression involves estrogen response elements (EREs), estrogen receptors (ERs), estrogen-related receptors (ERRs), retinoic acid receptors (RARs) and chicken ovalbumin upstream promoter transcription factor (Coup-TF) (88). Various *in vitro in vivo* experiments have demonstrated that estrus, menstruation, and pregnancy are associated with changes in the expression levels of the Lf gene. The concentration of Lf in breastmilk is related to the duration of lactation, with 5∼7 g/L of Lf in colostrum, then decreasing to 2∼3 g/L in mature milk (89). Using ultra performance liquid chromatography (UPLC), Cai et al. measured Lf levels in the breast milk of 248 lactating women from eight different regions in China, and the results showed that Lf levels gradually decreased from day 1 to day 30 (*p* < 0.01) and remained constant from day 31 to day 330 (90). Thus, Lf gene expression is also spatiotemporally specific.

**TABLE 2:** The content of Lf in the secretory fluid of human tissues.

Secretory fluid	Lf concentration	References
Breast milk	1∼3.2 mg/mL	(89)
Blood	0.1∼2.5 μg/mL	(3)
Bile	10∼40 μg/mL	(112)
Pancreatic juice	0.5 mg/mL	(113)
Gastric secretion	0.5∼1.0 mg/mL	(114)
Urine	1 μg/mL	(115)
Tear fluid	0.1∼2.2 mg/mL	(68)
Saliva	0.01∼0.05 mg/mL	(116)
Nasal secretion	0.1 mg/mL	(117)

The expression levels of Lf genes also vary considerably at the cellular level. Compared to normal cells, Lf expression is absent or reduced in leukemia and breast cancer cells (91). Through DNA sequencing of the human lactoferrin gene, Teng et al. found that this phenomenon is due to increased methylation of the CpG site of the lactoferrin gene and the presence of non-CpG methylation in tumor cells, i.e., an altered methylation pattern at the CpG site of the Lf gene in tumor cells (92). The regulation of Lf synthesis depends on the type of cells that produce Lf. The amount of Lf synthesized in the mammary gland is controlled by prolactin, while its synthesis in reproductive tissues is determined by estrogen. In addition, cell-derived miRNAs can also affect lactoferrin gene expression (93, 94). MiR-130a regulates C/EBP-ε protein expression in mouse and human granulocyte precursors, and overexpression downregulates C/EBP-ε and Lf (95). MiR-584 can upregulate lactoferrin receptor expression, and miR-214 directly targets Lf gene expression in cells (96, 97).

## 5. Discussion and prospect

Many studies have now shown that Lf is a reliable multifunctional protein with anti-inflammatory, antibacterial and antiviral effects. Because of its immune enhancing effects, it is also used in infant formulas. The immunomodulatory effect of Lf lies in its ability to modulate innate and adaptive immunity. One of the most interesting properties of Lf is that it can reach the nucleus as Lf possesses the ability to bind specifically to the LfR, which also gives Lf great potential to deliver macromolecules to the cytoplasm and nucleus, such as nucleic acids (siRNA, mRNA, shRNA, and DNA) (98). Lf is also used as a non-viral vector to deliver anti-cancer and anti-bacterial drugs for targeted therapeutic purposes. Currently researchers are investigating the role of Lf in post-operative infections such as organ transplants, prosthetic fittings, and skin grafts (99–102). Transgenic cows and goats are also being tried to produce recombinant hLf on a large-scale. Transgenic plants have improved antibacterial and disease resistance properties and can be used to the production of hypoallergenic lactoferrin. However, there is still a huge controversy about Genetically Modified (GM) foods, and the acceptance of GM foods has yet to be improved. In neurodegenerative diseases, Lf is specifically increased in saliva in patients with Alzheimer compared to Parkinson, dementia, and depression. So, we speculate that this may be related to a certain specific LfR. Lf also alters the tumor microenvironment and inhibits the metastasis of tumor cells. By evaluating effect of oral and intravenous (IV) administration of recombinant human Lf (rhLf) on blood cells, transcriptomic analysis showed that the majority of genes (72.8%) altered by oral rhLf are identical to IV treatment. Pathway mapping reveals a similar trend of upregulation of specific genes involved in oxidative stress and inflammatory responses in both treatment pathways, nevertheless the binding of Lf to cellular receptors and the mechanism of action of Lf in different diseases currently remain unknown. Furthermore, Lf also has different effects in diverse environments with various iron saturation, which is an area worth further exploring.

## Author contributions

XC contributed to the original draft, investigation, data curation, and drawing. KW, YW, and YHW contributed to the investigation. YR, QL, YZ, and ZC revised the manuscript. X-SC, ZY, and ZC contributed to the funding acquisition and resources. All authors have read and agreed to the published version of the manuscript.
